# Health-Related Quality of Life and Disease Acceptance Among Women with Breast Cancer Pre- and Post-Neoadjuvant Chemotherapy

**DOI:** 10.3390/cancers17030497

**Published:** 2025-02-02

**Authors:** Magdalena Konieczny, Jolanta Sawicka, Izabela Gąska, Elżbieta Kaczmar, Magdalena Babuśka-Roczniak, Dorota Bądziul

**Affiliations:** 1 Medical Institute, Jan Grodek State University in Sanok, 38-500 Sanok, Poland; 2 Department of Medical Biology, Institute of Medical Sciences, Medical College of Rzeszow University, Rejtana 16 C, 35-959 Rzeszów, Poland

**Keywords:** breast cancer, quality of life, acceptance, neoadjuvant chemotherapy

## Abstract

**Background/Objective:** The study aimed to evaluate disease acceptance and quality of life in women with breast cancer before and after receiving neoadjuvant chemotherapy. **Methods:** The study included 211 women diagnosed with breast cancer who underwent neoadjuvant treatment. The following measures were utilized: the EORTC QLQ-C30 and QLQ-BR23 modules for quality of life assessment, the Acceptance of Illness Scale (AIS) questionnaire for evaluating disease acceptance, and a proprietary questionnaire. Assessments were conducted one week before the first chemotherapy session and three weeks after completing the chemotherapy. Statistical analysis was performed using STATISTICA v. 13. **Results:** The respondents exhibited an average acceptance of their disease, both before (28.2 pts) and after chemotherapy (25.5 pts). A decline in disease acceptance was observed in nearly 59.2% of the patients following chemotherapy. Higher levels of disease acceptance were associated with a better quality of life. The quality of life for the studied women decreased after neoadjuvant chemotherapy, particularly in the following areas: physical functioning (*p* = 0.0000), social functioning (*p* = 0.0000), body image assessment (*p* = 0.0000), sexual satisfaction (*p* = 0.0000), nausea and vomiting (*p* = 0.0000), fatigue (*p* = 0.0000), loss of appetite (*p* = 0.0000), insomnia (*p* = 0.0000), pain (*p* = 0.0000), hair loss (*p* = 0.0000), and side effects of systemic treatment (*p* = 0.0000). **Conclusions:** Post-neoadjuvant chemotherapy, a decline in disease acceptance and quality of life was observed among women with breast cancer in comparison with their pre-treatment status. Higher levels of disease acceptance were associated with a better quality of life. These findings may facilitate the creation of a more tailored care approach for women during and after chemotherapy.

## Simple Summary

Breast cancer is the second most common malignant neoplasm globally, and it ranks fifth as a cause of death. One treatment option for breast cancer is systemic therapy, which can be administered before surgery—neoadjuvant chemotherapy. The use of this therapy is associated with adverse side effects. The study aimed to evaluate disease acceptance and quality of life in women with breast cancer before and after receiving neoadjuvant chemotherapy. The study included 211 women diagnosed with breast cancer. Assessments were conducted one week before the first chemotherapy session and three weeks after completing the chemotherapy. Post-neoadjuvant chemotherapy, a decline in disease acceptance and quality of life was observed among women with breast cancer in comparison with their pre-treatment status. Higher levels of disease acceptance were associated with a better quality of life. These findings may facilitate the creation of a more tailored care approach for women during and after chemotherapy.

## 1. Introduction

Recent epidemiological data indicate that the incidence of malignant tumors is steadily rising worldwide, with approximately 20 million new cases diagnosed each year. Consequently, around 10 million individuals die from malignant tumors annually [1]. Breast cancer is the second most common malignant neoplasm globally (the current incidence rate stands at 2.3 million cases per year), and it ranks fifth as a cause of death (nearly 700,000 fatalities each year) [2,3,4].

One treatment option for breast cancer is systemic therapy, which can be administered either before surgery (neoadjuvant chemotherapy) or following surgical treatment (adjuvant chemotherapy) [5,6]. Neoadjuvant chemotherapy is often used for patients with locally advanced or inoperable tumors to reduce tumor size and enhance the likelihood of successful surgical intervention. Recent studies indicate that even patients with non-advanced breast cancer can benefit from neoadjuvant chemotherapy as it may improve cosmetic outcomes and decrease the risk of postoperative complications [7,8]. The use of this therapy is associated with adverse side effects. It is important to note that breast cancer patients who receive neoadjuvant treatment will typically undergo additional stages of therapy, including surgery. The adverse effects of chemotherapy can impact the quality of life for these women and may lead to delays in subsequent procedures [9].

At present, quality of life assessment is widely regarded as the most sensitive and effective indicator of breast cancer outcomes. In breast cancer treatment, the emphasis is shifting toward not only extending lifespan but also preserving a high quality of life. Consequently, therapeutic intervention selection should prioritize treatment effectiveness and an optimal quality of life if possible [10,11].

The diagnosis of breast cancer is an incredibly challenging experience for women and their families. Moreover, oncological treatments, which are often aggressive, can significantly impact both the physical and emotional well-being of patients. Many patients fear the adverse effects of treatment, including a decline in physical abilities and changes to their appearance. This severe health challenge can lead to increased anxiety, emotional distress, and depression [12,13]. These factors can hinder a woman’s ability to accept her illness and adapt to her new circumstances. It is crucial to highlight the importance of disease acceptance in promoting mental and physical health. Acceptance can also serve as a motivator for pursuing treatment and improving overall quality of life [14,15].

Accordingly, studies focusing on the effects of this therapy on patients’ quality of life and their acceptance of the disease are fundamental. This information should play a crucial role in the treatment planning process. Quality of life assessments can provide valuable insights for optimizing the care of women post-chemotherapy, enabling a more personalized and effective approach and potentially resulting in improved overall well-being.

This study aimed to determine disease acceptance and quality of life in women with breast cancer pre- and post-neoadjuvant chemotherapy.

## 2. Materials and Methods

### 2.1. Organization and Selection of Patients for the Study

The study involved 211 women diagnosed with breast cancer. It was conducted at the Specialist Hospital, Podkarpacki Ośrodek Onkologiczny im. ks. B. Markiewicza, in Brzozów, Poland. Before the study began, all participants were informed about its purpose and assured of confidentiality, anonymity, and voluntary participation. They were also informed of their right to withdraw from the study at any stage. Eligible participants were women aged ≥18 with clinically confirmed stage I, II, or III breast cancer who had received neoadjuvant treatment, which included at least four cycles of chemotherapy based on taxanes and/or anthracyclines. During our study, no risk of adverse events resulting from the used treatment that would lead to discontinuation of this therapy were observed.

Approval for the study was granted by the director of the Ks. B. Markiewicz Specialist Hospital, Podkarpackie Oncological Centre. Additionally, the project received a positive opinion from the Bioethics Committee of Jan Grodek State University in Sanok (No. 3/2022).

### 2.2. Research Tools

The EORTC QLQ-C30 scale (European Organisation for Research and Treatment of Cancer Quality of Life Core Questionnaire) and its breast cancer-specific module, the EORTC QLQ-BR23 (EORTC BC-specific Quality of Life Questionnaire), were utilized to evaluate health-related quality of life [16]. Approval for using the QLQ-C30 and QLQ-BR23 questionnaires was granted by the Commission of the European Organisation for Quality of Life Research, based in Brussels. In Poland, the accuracy and reliability of the QLQ-C30 and QLQ-BR23 questionnaires were assessed, confirming their validity for evaluating the quality of life in cancer patients [17]. This validation was crucial in selecting these standardized research tools.

The QLQ-C30 questionnaire was used to assess cancer patients’ quality of life and health perception, covering physical, emotional, social, and cognitive functioning. It included an overall scale that evaluated health status and quality of life, five functional scales, three symptom scales, and six individual items (questions) that measured symptom intensity. The EORTC QLQ-BR23 scale assessed the quality of life in women with breast cancer and was a supplementary module to the QLQ-C30. The women assessed their body image, sexual functioning, sexual pleasure, prospects for the future, the effects of treatment, breast complaints, arm complaints, and feelings of sadness or stress related to hair loss.

A crude coefficient calculation was performed for each patient, followed by a linear transformation to derive a coefficient (score) that ranged from 0 to 100 points for both functional scales and individual symptoms. For the functional scales, a higher coefficient indicated a better level of functioning. Conversely, for symptom scales and individual symptoms, a higher coefficient reflected greater symptom severity, meaning the patient felt worse.

Acceptance of illness assessment was conducted using the standardized Acceptance of Illness Scale (AIS) questionnaire. The questions in the AIS questionnaire focused on the limitations imposed by the illness, lack of self-sufficiency, feelings of dependence on others, and diminished self-esteem. Each question used a five-point scale, where respondents indicated their current state of health by selecting a number: 1—strongly agree; 2—agree; 3—don’t know; 4—disagree; and 5—strongly disagree. A response of “strongly agree” indicated maladaptation to the illness, while a response of “disagree” suggested acceptance of it. The overall measure of acceptance was derived from the sum of all points, which could range from 8 to 40. A three-point range was created to categorize the degree of acceptance, resulting in a three-level adjective scale. A score between 8 and 18 signified no acceptance of the disease, a score from 19 to 29 indicated medium acceptance, and a score from 30 to 40 represented good acceptance.

Patients also filled out a short questionnaire regarding their socio-demographic data. The survey instruments were completed by the patients at two different times: one week before starting their first chemotherapy session and three weeks after completing the chemotherapy. Clinical data were obtained from their medical records.

### 2.3. Sample Size and Representativeness (Randomness)

It was assumed that all patients treated for breast cancer at the Podkarpackie Oncological Centre in Brzozów in 2024, meeting the inclusion criteria described in the methods and expressing informed consent to participate in the study, would be included in the study. It was assumed that patients from one of the hospitals in Poland constituted a representative group because uniform medical procedures related to the treatment of breast cancer were used throughout the country. Therefore, it could be assumed with great certainty that the selection used in the study guaranteed unbiased results and allowed conclusions to be drawn about the entire population of breast cancer patients in Poland (and beyond). Ultimately, the study included 211 women (which constituted 62% of all patients meeting the study criteria and treated in 2024 at the oncological center where the study was conducted). The sample size was verified using statistical tests, and as it turned out, the number of patients was sufficient to establish statistical significance of these correlations.

### 2.4. Statistical Analysis

Statistical analyses were performed using STATISTICA v. 13. The distribution of quality of life and disease acceptance measures in the overall population was described by determining selected descriptive statistics. The results were presented separately for the pre- and post-chemotherapy phases and the changes resulting from chemotherapy treatment. The Wilcoxon test was employed to evaluate the significance of changes in psychometric measures after chemotherapy. The results were considered statistically significant if the *p*-value was below 0.05 (such results were marked with an asterisk (*). For *p* < 0.01, double asterisks (**) were used, and for *p* < 0.001, triple asterisks (***) were indicated. Spearman’s rank correlation coefficient was utilized to assess the relationship between disease acceptance and overall quality of life (measured by QLQ-C30) and quality of life related to breast cancer (measured by QLQ-BR23). The choice of the nonparametric test was dictated by the lack of normality of the distribution of most of the analyzed measures—normality was verified using the Shapiro–Wilk test. The QLQ-C30 and QLQ-BR23 measures were characterized by strong skewness (in the case of functionality measures, it was right-sided asymmetry, and for the measures of complaints and symptoms, it was left-sided asymmetry).

### 2.5. Patient Characteristics

The mean life expectancy of the respondents was 56.2 years. The youngest patient was 32, and the oldest was 84 (Table 1).

Over half of the women surveyed, 55.5%, lived in rural areas, while the rest resided in urban areas, primarily in cities with populations ranging from 10,000 to 100,000 inhabitants. The study population was primarily composed of women in relationships (82.9%), with secondary or upper secondary education (43.1%), and in good financial standing (82.5%). Nearly half of the respondents, 48.8%, were economically inactive (Table 1).

## 3. Results

### 3.1. Acceptance of Illness

The overall disease acceptance level in the study group was 28.2 points pre-chemotherapy and 25.5 points post-chemotherapy. A statistically significant mean decrease of 2.7 points in AIS was observed (Wilcoxon test result: *p* = 0.0000 ***; Table 2).

It is essential to highlight that after chemotherapy, 59.2% of the respondents showed a decrease in their acceptance of the disease, as measured by the AIS scale. In contrast, 1/4 of the respondents exhibited a higher level of disease acceptance compared with their pre-treatment state, while 16.6% showed no change in their acceptance level (Table 3). The observed change was confirmed to be highly statistically significant, as determined by the Wilcoxon test (*p* = 0.0000 ***).

Table 4 presents the results of the AIS value classification on a three-level scale. Among women awaiting chemotherapy, 48.3% demonstrated a medium level of acceptance of their disease, while 43.1% showed a high level of acceptance. Conversely, 8.5% of the respondents indicated they did not accept their current condition. Following chemotherapy, the percentage of individuals with a high level of acceptance decreased from 43.1% to 29.4%. Additionally, the proportion of those who expressed no acceptance increased from 8.5% to 16.6%.

### 3.2. Quality of Life—EORTC QLQ-C30

The women in the study group awaiting chemotherapy rated their quality of life at 60.6 points. The highest ratings were for physical functioning (M = 81.3), fulfillment of social roles (M = 77.2), cognitive functioning (M = 76.5), and social functioning (M = 69.5). Among the symptom scales, the most frequently reported symptoms were insomnia (M = 34.4) and fatigue (M = 34.2).

When comparing the EORTC QLQ-C30 scores from the pre- and post-chemotherapy periods, a significant reduction in quality of life was observed after systemic treatment (Table 5). Decreased scores were noted for general health and quality of life, with declines in all functional scales and increased symptom severity. These differences were confirmed statistically (*p* < 0.001).

The most significant decreases in quality of life measures were observed in role fulfillment (−27.9) and physical functioning (−24.1). Conversely, among the symptoms, the most noteworthy increases were noted in nausea and vomiting (+28.9), fatigue (+27.0), lack of appetite (+25.6), insomnia (+20.7), and pain (+20.1). Details are presented in Table 5.

### 3.3. Quality of Life QLQ-BR23

An analysis of the women’s functional quality of life scales using the EORTC QLQ-BR23 questionnaire revealed that the subjects scored highest on body image (M = 69.8) and lowest on sexual functioning (M = 27.8) before chemotherapy. It is important to note that despite the low scores in the sexual functioning category among all participants, sexually active patients reported a sexual satisfaction score of M = 46.0. Additionally, the women in the study expressed concerns about their prospects, with a score of M = 40.8.

After chemotherapy, significant changes were observed in the functional and symptom scales of the EORTC QLQ-BR23 questionnaire. All functional scale scores decreased while symptom severity increased. The differences between the QLQ-BR23 measures before and after chemotherapy were statistically significant, with *p*-values from the Wilcoxon test being less than 0.001. The most notable differences in quality of life scores were in body image (−21.6) and sexual satisfaction (−17.3). For symptom scales, the most significant increases were seen in the effects of hair loss (+57) and side effects of systemic treatment (+24.6) (Table 6).

The correlations between the disease acceptance measure and overall quality of life (QLQ-C30) and disease-related quality of life (QLQ-BR23) were analyzed. These correlations were evaluated separately for the pre- and post-chemotherapy surveys, with detailed results in Table 7 and Table 8. Statistically significant correlations were found between AIS and nearly all QLQ-C30 measures. Before chemotherapy, the strongest correlation among the quality of life measures was between disease acceptance and overall quality of life (*r*_S_ = 0.41). This positive correlation indicates that higher levels of disease acceptance are associated with a higher quality of life. Regarding measures of discomfort, the strongest observed correlation was between financial difficulties and disease acceptance (*r*_S_ = −0.32). After chemotherapy, financial problems seemed to play a relatively smaller role compared with physical complaints.

Results similar to those observed in the correlation between acceptance of illness and the QLQ-C30 were also found with the QLQ-BR23 measures in relation to the AIS. Among the functional scales, the strongest correlation was observed between self-perception and the AIS scale (*r*_S_ = 0.31). After chemotherapy, no significant correlation with the AIS was found for the sexual satisfaction scale (*r*_S_ = 0.02) or the sexual functioning scale (*r*_S_ = 0.13) (Table 8). Furthermore, it is important to note that the correlations between the QLQ-C30 and QLQ-BR23 measures and the AIS were generally weak or very weak for most measures. The strength of these correlations was consistent both before and after chemotherapy.

## 4. Discussion

In recent years, neoadjuvant chemotherapy has become a standard treatment for breast cancer. This therapy positively impacts the success of breast-conserving surgery and improves the long-term prognosis for patients [18,19]. Nevertheless, it also comes with side effects that can diminish the quality of life. It is essential to understand that neoadjuvant chemotherapy typically represents one of the initial phases of treatment, which may be followed by surgery and additional therapies, such as supplementary chemotherapy or radiotherapy [20]. Given this context, it is crucial to recognize the potential changes in various aspects of quality of life. The side effects of treatment—physical, psychological, and social—should be considered when planning care and support for patients both during and after their treatment. Breast cancer can be a significant source of stress, and patients’ responses throughout their illness may be influenced by various factors, including their level of acceptance of the illness [21].

Our study demonstrated that the women surveyed exhibited an average level of disease acceptance, both before (28.2) and after chemotherapy (25.5). Another study conducted in Poland also assessed disease acceptance among women with breast cancer undergoing outpatient chemotherapy and reported a mean score of 28.45 on the AIS scale, indicating an average level of acceptance [22]. However, it is important to note that after chemotherapy, nearly 60% of the women in our study showed a decrease in disease acceptance as measured by the AIS. This finding highlights a significant need for interventions aimed at increasing the level of disease acceptance. Higher acceptance of the illness can enhance motivation to combat the disease and improve health management. Additionally, adaptability to dysfunctions caused not only by the disease but also by its treatment tends to improve with greater acceptance. Our study also found that illness acceptance had a positive impact on the quality of life for the women involved. These results align with the other literature in this area. For instance, a study by Lee et al. identified negative illness perceptions among patients currently undergoing chemotherapy, as well as those who had completed treatment. Furthermore, negative illness perceptions were associated with a direct decline in feelings of well-being [23].

Monitoring the quality of life during cancer treatment, particularly for breast cancer, is a priority as diagnosis and treatment can significantly affect women’s functioning. Data from the literature indicate that in clinical trials, patient-reported outcomes can provide information on the side effects of treatment [24]. However, incorporating these findings into daily oncological practice poses challenges due to difficulties in interpreting study results. Currently, some scientific reports are working to establish reference values for specific quality of life scales. For instance, Giesinger et al. have defined thresholds for clinical importance (TCIs) for both the functional and symptomatic scales of the EORTC QLQ-C30. In this context, a score below the TCI for functional scales indicates a clinically relevant problem, while a score above the TCI for symptom scales suggests the presence of such a problem [25].

Our study revealed that women with breast cancer awaiting chemotherapy reported a quality of life score of 60.6 points. However, after undergoing chemotherapy, this score significantly dropped to 42.3. Notably, even before chemotherapy, the scores for physical functioning and social functioning were already below the established clinical significance thresholds. This indicates a substantial impairment in women’s functioning in these areas from the outset [25]. Additionally, both scales showed the most significant decline in scores after chemotherapy. The neoadjuvant treatment led to a decrease in quality of life across all functional and symptomatic scales. Compared with the clinical importance thresholds established by Geisinger et al., these results indicate a severe clinical concern [25]. The most pronounced differences were observed in the symptom scales, which assessed the severity of nausea and vomiting, fatigue, loss of appetite, insomnia, and pain.

The study conducted by Ding et al. evaluated the impact of neoadjuvant chemotherapy on respiratory function in breast cancer patients [26]. The study included patients who underwent four cycles of neoadjuvant chemotherapy using a regimen of cyclophosphamide, epirubicin, and 5-fluorouracil (CEF regimen). The findings revealed a decline in overall health status and quality of life (85.9 vs. 74.6). Furthermore, there was an increase in symptoms such as dyspnea (2.8 vs. 11.1) and fatigue (3.4 vs. 18.4). Studies by other authors have also demonstrated a decrease in the quality of life for women following chemotherapy, attributed to heightened experiences of pain, nausea, vomiting, dyspnea, and fatigue [27,28].

Patients’ quality of life tends to decline during chemotherapy due to the toxic effects of the medications used in this treatment. However, many side effects diminish over time, leading to an improvement in quality of life. Therefore, it would be beneficial to conduct further studies assessing the quality of life in the same group of patients after an extended period following chemotherapy.

In our study, the quality of life was most significantly impacted by increased symptoms such as nausea and vomiting, fatigue, lack of appetite, insomnia, and pain. The most significant differences were observed between pre- and post-treatment measurements in these categories. A study conducted by Lee et al. found that 48.5% of patients undergoing neoadjuvant chemotherapy experienced nausea and vomiting, with 42.5% experiencing delayed nausea and vomiting [29].

In a study conducted by Takada et al. involving women with breast cancer, patients were divided into two groups based on their overall quality of life scores before receiving neoadjuvant chemotherapy: those with lower scores and those with higher scores. The researchers found significant differences between the two groups in various areas, including physical functioning, pain perception, and sexual aspects. Women with lower overall quality of life scores exhibited lower values on these scales. However, after the administration of neoadjuvant chemotherapy, no significant differences were observed between the two groups in these subscales [30]. It is important to note that although no differences in quality of life were identified between the two groups after treatment, the overall quality of life for all participants declined following neoadjuvant chemotherapy. The findings from our study also support this decline.

In our study, we found that fatigue significantly increased after chemotherapy. It is noteworthy that there have been few measurements of fatigue and quality of life during neoadjuvant chemotherapy in breast cancer patients. One study by Pelzer et al. indicated that all dimensions of fatigue worsened to a clinically relevant degree, with physical fatigue increasing the most and mental fatigue the least. Regarding quality of life, the most significant deterioration was observed in physical and functional well-being [31]. Our findings corroborate this as we observed the greatest decline in the quality of life related to the physical functioning scale. Also, Pelzer et al. demonstrated that patients who started the study with higher levels of fatigue experienced lower levels of fatigue intensity during chemotherapy. The authors also highlighted that cancer-related fatigue during chemotherapy is an unmet clinical need. Patients with early-stage breast cancer begin neoadjuvant chemotherapy with fatigue levels comparable to those of the general population, but they typically end the treatment with a critically high level of cancer-related fatigue (CRF) [31].

## 5. Limitations of the Study

The study had some limitations. Its findings were restricted to a single clinical center, thus limiting the sample’s representativeness. Nevertheless, this constraint also presents a valuable opportunity for expeditious implementation of interventions designed to improve the quality of life of women with breast cancer during and after chemotherapy. Given the sample size of the study group, additional research is warranted both retrospectively and prospectively. In addition, it is advisable to conduct a long-term follow-up to assess whether changes in acceptance of the disease and quality of life are permanent or temporary.

## 6. Conclusions

1. The women studied exhibited an average level of disease acceptance, both before (28.2) and after chemotherapy (25.5). Notably, almost 60% of patients experienced a decline in disease acceptance, as indicated by the AIS scale, after undergoing chemotherapy.
2. Disease acceptance positively affected the quality of life for the women studied. A higher level of acceptance was associated with an improved quality of life.
3. Following neoadjuvant chemotherapy, the quality of life for the studied women decreased. The most significant declines were observed in the following functional scales: physical functioning, social functioning, body image assessment, and sexual satisfaction. Additionally, notable decreases were reported in symptom scales, including nausea and vomiting, fatigue, lack of appetite, insomnia, pain, hair loss, and side effects from systemic treatments.
4. The findings from this study can guide the development of a more personalized approach to care for women during and after chemotherapy, ultimately aiming to enhance their quality of life.

## Figures and Tables

**Table 1:** Socio-demographic characteristics of respondents.

Age (Years)							
Mean	Median	Std. Dev.	Low Quartile	Upper Quartile	Min.		Max.
56.2	56	11.0	48	65	32		84
**Characteristics**				**N**		**%**	
**Place of residence**							
Countryside				117		55.5%	
City up to 20,000 inhabitants				36		17.1%	
City of 10,000–100,000 inhabitants				51		24.2%	
City of over 100,000 inhabitants				7		3.3%	
**Marital status**							
Single				36		17.1%	
In a relationship				175		82.9%	
**Education**							
Primary/secondary				11		5.2%	
Basic vocational				44		20.9%	
Secondary/upper-secondary				91		43.1%	
Bachelor’s/master’s degree				65		30.8%	
**Financial standing**							
Very good				11		5.2%	
Good				174		82.5%	
Bad				26		12.3%	
**Professional activity**							
Physical work				37		17.5%	
Office work				67		31.8%	
Retirement/invalidity pension				39		18.5%	
Jobless				64		30.3%	
Other sources of livelihood				4		1.9%	

**Table 2:** Mean acceptance level of the disease in the studied group of women pre- and post-chemotherapy.

AIS	Mean (with 95% CI)	Median	Std. Dev.	Min.	Max.
Pre-chemotherapy	28.2 (27.2; 29.2)	28	7.1	8	40
Post-chemotherapy	25.5 (24.5; 26.5)	26	7.1	8	40
Change (*p* = 0.0000 ***)	−2.7 (−3.7; −1.7)	−2	7.3	−24	17

*p*—assessment of the significance of changes between studies using the Wilcoxon test; ***—*p* < 0.001.

**Table 3:** Changes in disease acceptance post-chemotherapy vs. pre-chemotherapy.

Disease Acceptance Post-Chemotherapy vs. Pre-Chemotherapy (*p* = 0.0000 ***)	Number	Percentage
Decrease	125	59.2%
No change	35	16.6%
Height	51	24.2%

*p*—assessment of the significance of changes between studies using the Wilcoxon test; ***—*p* < 0.001.

**Table 4:** Women’s illness acceptance level, based on the AIS scale, in the study cohort pre- and post-chemotherapy.

Illness Acceptance Level	Pre-Chemotherapy		Post-Chemotherapy	
	*N*	%	*N*	%
None	18	8.5%	35	16.6%
Mean	102	48.3%	114	54.0%
High	91	43.1%	62	29.4%

**Table 5:** Quality of life assessment of women with breast cancer pre- and post-chemotherapy: categories related to QLQ-C30.

QLQ-C30	Mean (with 95% CI)			*p*
	Pre-Chemotherapy	Post-Chemotherapy	Change	
Functional scales				
Global health status	60.6 (57.9; 63.2)	42.3 (39.8; 44.9)	−18.2 (−21.6; −14.9)	0.0000 ***
Physical functioning	81.3 (78.5; 84.1)	57.2 (54.2; 60.2)	−24.1 (−27.5; −20.7)	0.0000 ***
Role functioning	77.2 (73.6; 80.8)	49.3 (45.5; 53.1)	−27.9 (−32.2; −23.6)	0.0000 ***
Emotional functioning	63.7 (60.5; 66.9)	49.2 (45.6; 52.9)	−14.4 (−18.3; −10.6)	0.0000 ***
Cognitive functioning	76.5 (73.3; 79.6)	58.2 (54.5; 61.9)	−18.2 (−22.1; −14.4)	0.0000 ***
Social functioning	69.5 (65.5; 73.5)	49.7 (45.7; 53.7)	−19.8 (−24.3; −15.3)	0.0000 ***
Symptom scales				
Fatigue	34.2 (31.3; 37.1)	61.2 (58.1; 64.3)	27.0 (23.1; 30.9)	0.0000 ***
Nausea and vomiting	11.6 (9.2; 14.0)	40.5 (36.3; 44.8)	28.9 (24.7; 33.1)	0.0000 ***
Pain	23.1 (20.2; 26.1)	43.3 (39.5; 47.1)	20.1 (16.3; 24.0	0.0000 ***
Dyspnea	16.1 (12.9; 19.3)	33.2 (28.9; 37.4)	17.1 (13.2; 20.9)	0.0000 ***
Insomnia	34.4 (30.6; 38.3)	55.1 (50.8; 59.5)	20.7 (16.2; 25.2)	0.0000 ***
Appetite loss	23.2 (19.3; 27.2)	48.8 (44.4; 53.2)	25.6 (20.5; 30.7)	0.0000 ***
Constipation	17.5 (14.5; 20.6)	31.9 (28.2; 35.6)	14.4 (10.5; 18.3)	0.0000 ***
Diarrhea	11.4 (8.5; 14.2)	26.1 (22.0; 30.1)	14.7 (10.8; 18.6)	0.0000 ***
Financial difficulties	24.5 (20.4; 28.6)	38.4 (34.1; 42.7)	13.9 (9.6; 18.2)	0.0000 ***

*p*—assessment of the significance of changes between studies using the Wilcoxon test; ***—*p* < 0.001

**Table 6:** Quality of life assessment of women with breast cancer pre- and post-chemotherapy: categories related to QLQ-BR23.

QLQ-BR23	Mean (with 95% CI)			*p*
	Pre-Chemotherapy	Post-Chemotherapy	Change	
Functional scales				
Body image	69.8 (65.9; 73.7)	48.2 (44.5; 51.9)	−21.6 (−25.7; −17.5)	0.0000 ***
Sexual functioning	27.8 (24.2; 31.4)	15.7 (12.8; 18.6)	−12.1 (−15.9; −8.3)	0.0000 ***
Sexual pleasure	46.0 (40.9; 51.1)	29.0 (23.8; 34.3)	−17.3 (−24.8; −9.8)	0.0002 ***
Future perspective	40.8 (36.3; 45.2)	26.9 (23.0; 30.7)	−13.9 (−18.4; −9.4)	0.0000 ***
Symptom scales				
Systematic therapy side effects	26.0 (23.3; 28.7)	50.6 (48.1; 53.2)	24.6 (21.1; 28.2)	0.0000 ***
Breast symptoms	25.7 (22.8; 28.5)	32.8 (29.2; 36.4)	7.1 (3.7; 10.6)	0.0002 ***
Arm symptoms	18.5 (15.9; 21.0)	29.8 (26.4; 33.2)	11.3 (8.1; 14.6)	0.0000 ***
Upset by hair loss	3.0 (1.7; 4.3)	60.0 (55.4; 64.6)	57.0 (52.2; 61.9)	0.0000 ***

*p*—assessment of the significance of changes between studies using the Wilcoxon test; ***—*p* < 0.001.

**Table 7:** Correlations of QLQ-C30 measures with AIS.

Measures of QLQ-C30	Correlations of QLQ-C30 Measures with AIS	
	Pre-Chemotherapy	Post-Chemotherapy
Functional scales		
Global health status	0.41 (*p* = 0.0000 ***)	0.47 (*p* = 0.0000 ***)
Physical functioning	0.23 (*p* = 0.0009 ***)	0.29 (*p* = 0.0000 ***)
Role functioning	0.28 (*p* = 0.0000 ***)	0.30 (*p* = 0.0000 ***)
Emotional functioning	0.26 (*p* = 0.0001 ***)	0.43 (*p* = 0.0000 ***)
Cognitive functioning	0.32 (*p* = 0.0000 ***)	0.24 (*p* = 0.0006 ***)
Social functioning	0.36 (*p* = 0.0000 ***)	0.35 (*p* = 0.0000 ***)
Symptom scales		
Fatigue	−0.23 (*p* = 0.0006 ***)	−0.26 (*p* = 0.0001 ***)
Nausea and vomiting	−0.14 (*p* = 0.0469 *)	−0.25 (*p* = 0.0002 ***)
Pain	−0.23 (*p* = 0.0007 ***)	−0.18 (*p* = 0.0089 **)
Dyspnea	−0.18 (*p* = 0.0088 **)	−0.24 (*p* = 0.0004 ***)
Insomnia	−0.11 (*p* = 0.1138)	−0.25 (*p* = 0.0003 ***)
Appetite loss	−0.22 (*p* = 0.0015 **)	−0.24 (*p* = 0.0003 ***)
Constipation	−0.20 (*p* = 0.0044 **)	−0.11 (*p* = 0.1187)
Diarrhea	−0.18 (*p* = 0.0095 **)	−0.17 (*p* = 0.0137 *)
Financial difficulties	−0.32 (*p* = 0.0000 ***)	−0.22 (*p* = 0.0014 **)

*p*—assessment of the significance of changes between studies using the Wilcoxon test; * *p* < 0.05; ** *p* < 0.01; ***—*p* < 0.001.

**Table 8:** Correlations of QLQ-BR23 measures with AIS.

QLQ-BR23 Measures	Correlations of QLQ-BR23 Measures with AIS	
	Pre-Chemotherapy	Post-Chemotherapy
Functional scales		
Body image	0.31 (*p* = 0.0000 ***)	0.31 (*p* = 0.0000 ***)
Sexual functioning	0.20 (*p* = 0.0033 **)	0.13 (*p* = 0.0564)
Sexual pleasure	0.29 (*p* = 0.0007 ***)	0.02 (*p* = 0.8268)
Future perspective	0.17 (*p* = 0.0152 *)	0.22 (*p* = 0.0014 **)
Symptom scales		
Systematic therapy side effects	−0.31 (*p* = 0.0000 ***)	−0.27 (*p* = 0.0001 ***)
Breast symptoms	−0.17 (*p* = 0.0123 *)	−0.18 (*p* = 0.0080 **)
Arm symptoms	−0.20 (*p* = 0.0042 **)	−0.18 (*p* = 0.0074 **)
Upset by hair loss	−0.08 (*p* = 0.2637)	−0.16 (*p* = 0.0190 *)

*p*—assessment of the significance of changes between studies using the Wilcoxon test; * *p* < 0.05; ** *p* < 0.01; ***—*p* < 0.001.

## Data Availability

All data generated or analyzed during this study are included in this published article.
